# Exploring the Processing Paradigm of Input Data for End-to-End Deep Learning in Tool Condition Monitoring

**DOI:** 10.3390/s24165300

**Published:** 2024-08-15

**Authors:** Chengguan Wang, Guangping Wang, Tao Wang, Xiyao Xiong, Zhongchuan Ouyang, Tao Gong

**Affiliations:** 1 Institute of Intelligent Manufacturing Technology, Shenzhen Polytechnic University, Shenzhen 518055, China; wangchengguan@szpu.edu.cn; 2 AVIC Changhe Aircraft Industry (Group) Corporation Ltd., Jingdezhen 333002, China; wanggp005@avic.com (G.W.); xiongxy003@avic.com (X.X.); ouyzc@avic.com (Z.O.); 3 Institute of Ultrasonic Technology, Institute of Intelligent Manufacturing Technology, Shenzhen Polytechnic University, Shenzhen 518055, China

**Keywords:** tool condition monitoring, end-to-end, deep learning, input data, convolutional neural network, bidirectional long short-term memory

## Abstract

Tool condition monitoring technology is an indispensable part of intelligent manufacturing. Most current research focuses on complex signal processing techniques or advanced deep learning algorithms to improve prediction performance without fully leveraging the end-to-end advantages of deep learning. The challenge lies in transforming multi-sensor raw data into input data suitable for direct model feeding, all while minimizing data scale and preserving sufficient temporal interpretation of tool wear. However, there is no clear reference standard for this so far. In light of this, this paper innovatively explores the processing methods that transform raw data into input data for deep learning models, a process known as an input paradigm. This paper introduces three new input paradigms: the downsampling paradigm, the periodic paradigm, and the subsequence paradigm. Then an improved hybrid model that combines a convolutional neural network (CNN) and bidirectional long short-term memory (BiLSTM) was employed to validate the model’s performance. The subsequence paradigm demonstrated considerable superiority in prediction results based on the PHM2010 dataset, as the newly generated time series maintained the integrity of the raw data. Further investigation revealed that, with 120 subsequences and the temporal indicator being the maximum value, the model’s mean absolute error (MAE) and root mean square error (RMSE) were the lowest after threefold cross-validation, outperforming several classical and contemporary methods. The methods explored in this paper provide references for designing input data for deep learning models, helping to enhance the end-to-end potential of deep learning models, and promoting the industrial deployment and practical application of tool condition monitoring systems.

## 1. Introduction

Prognostics and health management (PHM), widely applied in manufacturing systems to monitor system operating conditions and ensure the reliability of key components, are reshaping modern manufacturing through the continuous development of intelligent manufacturing technology [1]. Cutting, as an important manufacturing method in the machining process, uses tools to perform machining operations. Tool wear is inevitable under the action of thermodynamic coupling, particularly for hard-to-cut materials like aluminum alloys, widely used in aerospace manufacturing. Tool wear directly affects machining accuracy, surface quality, and workpiece production efficiency. Studies have shown that accurate monitoring of tool conditions can reduce downtime by 75%, increase productivity by 65%, and save maintenance costs by 30% [2,3]. Therefore, tool condition monitoring (TCM) technology is an indispensable part of intelligent manufacturing.

TCM methods are divided into direct measurement methods and indirect measurement methods [4]. Direct measurement methods acquire tool wear information offline through microscopy and camera capture. However, due to their sensitivity to cutting fluid, chips, vibration, and various other environmental interferences, direct measurement methods only function when the system is offline, making them incapable of real-time monitoring of tool wear status [5,6]. Indirect measurement methods use various sensors to collect signals such as cutting force, vibration, acoustic emission, and current in real time, then use intelligent algorithms to establish a nonlinear relationship between sensor signals and tool wear, obtaining tool wear values in real time [7]. Given the actual production scenarios and rhythms, indirect measurement methods are more suitable for online tool wear prediction [8].

**Figure 1:**
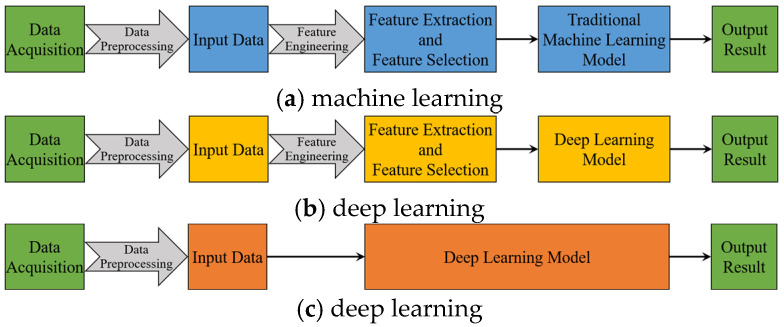
Stages involved in both traditional machine learning and deep learning.

In recent years, indirect measurement of tool wear has become a research hotspot in the TCM field [9]. Early studies extensively researched TCM based on traditional machine learning (ML) [10,11,12,13,14,15,16], as illustrated by the technical route in Figure 1a. First, preprocess the collected raw signals by removing invalid values and denoising to obtain input data, then perform feature engineering to extract and select features related to tool wear, and finally use ML models to predict tool wear amounts. For instance, Gomes et al. [17] analyzed the vibration and sound signals in both time and frequency domains and then used the recursive feature elimination (RFE) method to choose features that were then fed into a support vector machine (SVM). This gave them a classification accuracy of up to 97.54%. Li et al. [18] rearranged signals based on domain knowledge into features related to spindle speed and machine tool structure and embedded them in a random forest (RF) algorithm, improving tool wear status prediction accuracy to 84.1%. Dhobale et al. [19] compared different wavelet families with the naïve Bayes and Bayes net classifiers and found that the Sym5 wavelet with the naïve Bayes classifier works best for finding damage in the end milling tool. Despite the effectiveness of these traditional ML model-based studies in tool wear monitoring, they require complex signal processing techniques and feature engineering that require expert experience and prior knowledge, increasing monitoring difficulty and reducing efficiency. As the cornerstone of traditional ML applications, feature engineering is both difficult and expensive [20]. Unfortunately, sensor signal data are essentially time series, and the statistical features obtained through feature engineering cannot fully capture time series trend changes [21,22]. Faced with the large amount of signal data provided in the intelligent manufacturing era, traditional ML model-based TCM is becoming increasingly impractical [8].

Deep learning (DL) offers a new solution to these challenges with its end-to-end learning model. Since Hinton et al. [23] found out in 2006 that deep hidden layer neural networks can learn underlying features in data, DL models with multiple hidden layers of deep neural networks have shown the ability to learn new features as needed. Layer-by-layer feature learning in DL models can find hidden features in data because the networks are more complex. This lets them understand complex, nonlinear relationships from signals that have not been processed much or at all, which makes predictions much more accurate and efficient [24,25]. But because sensor signal data are often noisy and there are not enough data samples in real life, many studies still take features from raw signal data before teaching DL models how to predict tool wear [26,27,28,29,30,31,32], as shown in Figure 1b. This is similar to the traditional TCM method shown in Figure 1a. For example, Shah et al. [33] constructed the scalograms of signals from Morlet wavelets and later constructed feature vectors with image quality parameters. Then they used the feature vectors to train different long short-term memory (LSTM) variants and concluded that the stacked LSTM model predicted the tool wear best. Liu et al. [34] took time-domain features from current signals and used monotonicity to choose which features to use. They then put these features into a bidirectional long short-term memory (BiLSTM) model to guess how long tools will still be useful. Barrena et al. [35] extracted different features from all signals and evaluated the optimum signals using recursive feature elimination (RFE). To predict tool wear, they used bidirectional recurrent neural networks (BRNNs) as regressive models. Huang et al. [36] extracted multi-domain features from cutting force and vibration signals, including time-domain, frequency-domain, and time–frequency-domain features, and designed a deep convolutional neural network (CNN) based on multi-domain feature fusion to establish a mapping relationship between these multi-domain features and a real-time tool. Bazi et al. [37] suggested a new way to break down signals called variational mode decomposition (VMD). They used a CNN-BiLSTM hybrid model to look through VMD collaborative data, which made TCM more accurate. Zhang et al. [38] came up with a way to deal with uneven input data features by using the Hurst index to divide sensor data into small groups and combined signal segmentation with DL algorithms. This method was 87.3% accurate at predicting what would happen next. Sayyad et al. [39] noted that the provision of well-established data to the prediction model determines the performance of tool wear prediction and, as a result, they focused on selecting the appropriate and optimal features from all features, which significantly reduces the complexity of the raw data for analysis. Given the close integration of feature engineering and DL, Wang et al. [40] even discussed solutions for feature selection and hyperparameter tuning in tool wear monitoring systems. Although these TCM methods combining DL models with feature engineering have made significant progress in tool wear prediction, it is clear that DL methods are merely replacements for traditional ML models without fully exploiting the strong adaptive feature extraction and nonlinear mapping capabilities of DL models [41].

Luckily, more researchers are becoming aware of this problem and are starting to try directly putting raw data that has already been processed into DL models in TCM applications [21,42,43,44,45,46,47,48], which avoids the difficulties and restrictions that come with feature engineering. As shown in the technical route in Figure 1c, this provides a complete suite for the prediction process at one time. Zhao et al. [49] first explored directly applying raw time series data to LSTM models to predict tool wear. They divided the raw data sequence into 100 sections, keeping the maximum value of each section to form a new time step for the LSTM. Marani et al. [50] tested the LSTM model using spindle motor current signals and found that the most accurate model contained two layers and eight hidden units. Kim et al. [51] used the sliding-window preprocessing method to convert the raw data into multiple subsequences with identical lengths, and then proposed a deep multi-scale CNN (DMSCNN) to extract multi-scale information from the preprocessed data. Similarly, Jeon and Rhee [52] aimed to predict tool wear using a Seq2Seq model, applying the sliding-window method to preprocess the raw data and make them suitable for model learning. After processing the raw signal data, they determined the length of the sequence and implemented a downsampling strategy. Yin et al. [53] used a CNN to pull out features from processed raw data and then combined those using deep generalized canonical correlation analysis (DGCCA) and attention mechanisms, which they said gave them a 95.6% success rate. Chan et al. [54] used a hybrid model of a CNN and LSTM to solve the problem of high precision in predicting tool wear that comes from the complex spatiotemporal properties of multi-sensor data. This model effectively extracted the two-dimensional correlation of multiple sensors and the temporal correlation of time series without the need for manual extraction. In their study, segmented feature extraction proved to be more effective at capturing the feature information from the raw data than holistic feature extraction. Furthermore, Nie et al. [55] combined attention mechanisms with the CNN-BiLSTM hybrid model to selectively study important degradation features of tool wear, improving tool condition prediction accuracy. As for their input data, the raw data were first intercepted to obtain a stable segment and then downsampled. Recently, Ma et al. [56] proposed an end-to-end tool wear condition monitoring algorithm that combines a CNN and Transformer. The former extracts local features from the raw signal preprocessed by the sliding-window method, while the latter captures the global feature relationship. These studies fully exploit the end-to-end advantages of DL without the need for complex feature engineering, simplifying the data process, making model construction more straightforward, and promoting TCM towards more efficient and automated development.

Gradual research reveals that the tool wear process is gradual, random, nonlinear, and heavily dependent on temporal characteristics [42,57]. Various types of sensors typically are equipped to monitor the tool machining process, leading to complex spatiotemporal correlations between the collected multi-channel sensor signal data and tool wear [58]. A single prediction model cannot fully capture this intricate mapping relationship. As a result, current TCM research based on DL models mostly adopts more complex hybrid models, combining CNN and recurrent neural network (RNN) models, which have become the latest trend due to their ability to fully exploit data temporal and spatial features [59,60,61,62]. However, in order to apply end-to-end DL methods in TCM, it is essential not only to design a reasonable DL model but also to feed sufficient information related to tool wear into the DL model. If the complete time-domain signal is used as the model input, it significantly reduces the model’s training speed and increases the equipment’s computational burden. However, simply ignoring some signals may lead to a substantial loss of information related to tool wear due to the continuous nature of signal acquisition [63]. A thorough look at the existing research mentioned above makes it clear that most of the studies are focused on advanced, complicated algorithms to improve predictive performance of TCM methods. Not much thought is given to how to effectively feed large amounts of structurally complex, feature-diverse, multi-sensory raw data into DL models. Even more unfortunately, there is no clear reference standard on how to process multi-sensor raw data into input data directly usable for training DL models while reducing the input data scale and maintaining adequate temporal interpretation of tool wear. Existing research is based on empirical design.

Based on this, this paper explored the data processing methods for converting raw data into input data for DL models used in TCM, referred to as input paradigms. First, three novel input paradigms were designed: the downsampling paradigm, the periodic paradigm, and the subsequence paradigm. Then an improved CNN-BiLSTM hybrid model was designed to validate the performance of various input paradigms. The research results provided references and guidance for designing input data for DL models, helping to enhance the real-time effectiveness of TCM methods based on end-to-end DL models, and promoting the industrial deployment and practical application of TCM systems.

## 2. Methodology of Input Paradigm and Time Series Presentation

### 2.1. Problem Description

In the actual tool machining process, tool wear values cannot be collected in real time but are measured when the machine stops after completing the cutting process, while sensor signals monitoring tool condition are continuously collected in real time during the machining process. In this paper, “S” and “V” are used to represent the tool monitoring signals and tool wear values, respectively. The time taken to complete one cutting process can be regarded as a time step “T”. Thus, within “T”, a large amount of signal data “$S_{T}$” (recorded in matrix form) and a specific wear value “$V_{T}$” will be obtained, as shown in Equation (1).

(1) $$S_{T}={\left[\begin{array}{ccc} S_{11} & \cdots & S_{1c}\\ \vdots & \ddots & \vdots \\ S_{t1} & \cdots & S_{tc} \end{array}\right]}_{t\times c}\;,\;V_{T}$$

where t denotes both the number of sampling points and c the number of signal channels, also known as signal types. Assuming the entire machining process involves cutting operations, Equations (2) and (3) show the signal matrix obtained during the entire machining process and its corresponding wear values.

(2) $$S=\left\{S_{1},\;S_{2},\;\cdots \;{,\;S}_{T},\;\cdots {\;,\;S}_{M}\right\}$$

(3) $$V=\left\{V_{1},\;V_{2},\;\cdots \;{,\;V}_{T},\;\cdots \;{,\;V}_{M}\right\}$$

As shown in Equation (4), the multi-channel sensor signals and tool wear values collected here correspond one-to-one, forming a dataset that can be used as input for the tool wear prediction model to train and test the DL model.

(4) $$\left\{\left(S_{1},V_{1}\right),\left(S_{2},V_{2}\right),\cdots \left(S_{T},V_{T}\right),\cdots ,\left(S_{M},V_{M}\right)\right\}$$

Therefore, the tool wear prediction problem is transformed into a regression problem of time series data.

### 2.2. Design of Different Input Paradigms

As mentioned in the Introduction, this paper designs three different input paradigms, detailed as follows:

#### 2.2.1. Downsampling Paradigm

**Figure 2:**
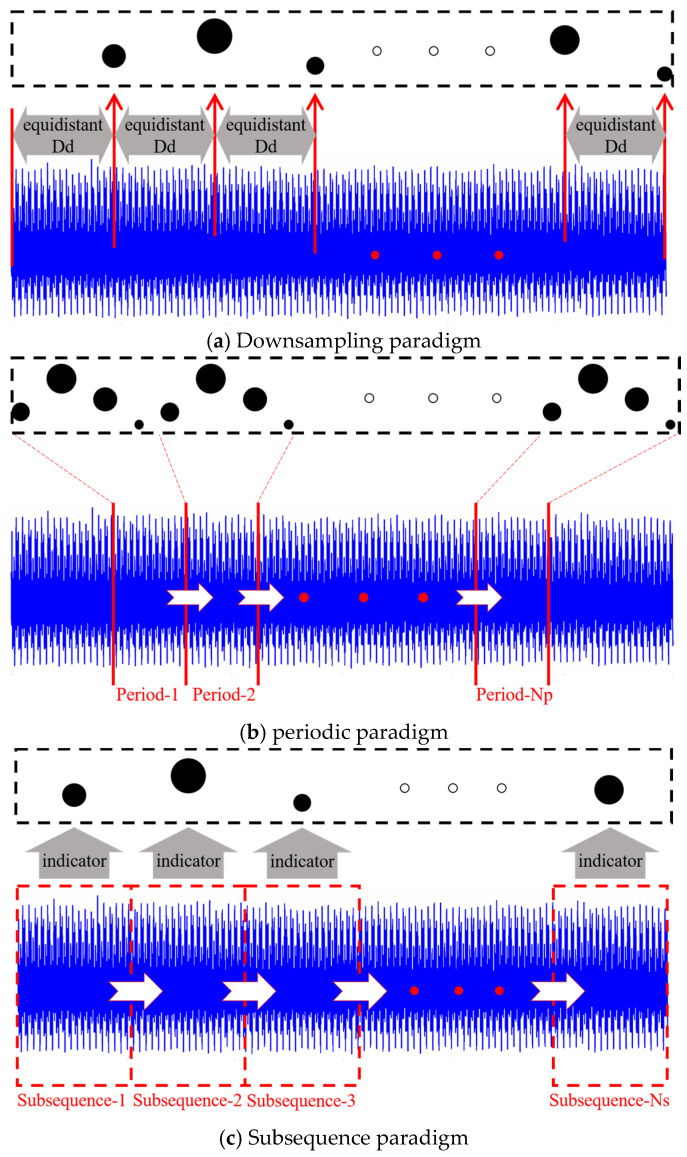
Schematic diagram of the three input paradigms proposed in this paper.

Downsampling is a well-established strategy that addresses large-scale datasets, reducing data scale and preventing the loss of key features [44,53,55]. Figure 2a illustrates its basic principle. The original data are processed at equal intervals of $N_{d}$, meaning one sampling point is retained for every $N_{d}$ sampling points, sequentially generating a new series. The downsampling rate then is the reciprocal of $N_{d}$. If the length of the original signal is N, then the length of the newly generated series is $N/N_{d}$. In the following, the downsampling paradigm processed at equal intervals of $N_{d}$ will be denoted as downsampling-$N_{d}$.

#### 2.2.2. Periodic Paradigm

The tool rotates at a high speed during the cutting process, which theoretically can be considered a periodic circular motion. If the spindle speed of the machine tool is n and the signal sampling rate is $f_{s}$, then the number of sampling points $N_{c}$ collected per rotation of the tool is:

(5) $$N_{c}=f_{s}/\left(n/60\right)$$

If the number of selected periods is $N_{p}$, then the sampling points of these cycles can be sequentially spliced into a new sequence with a length of $N_{p}\cdot N_{c}$. Figure 2b illustrates this process. In the following, the periodic paradigm selecting $N_{p}$ periods will be denoted as periodic-$N_{p}$.

#### 2.2.3. Subsequence Paradigm

Figure 2c illustrates the basic principle of the subsequence paradigm. Specifically, the collected raw signal data are first evenly divided into $N_{s}$ subsequences along the time dimension. Then, the temporal indicator (such as maximum value, minimum value, and mean value) of each subsequence is calculated. Finally, these indicator values are sequentially connected to form a new time series. Obviously, the length of this new series is the number of subsequences, $N_{s}$. In the following, the subsequence paradigm using $N_{s}$ subsequences will be denoted as subsequence-$N_{s}$.

### 2.3. PHM2010 Dataset Description

**Figure 3:**
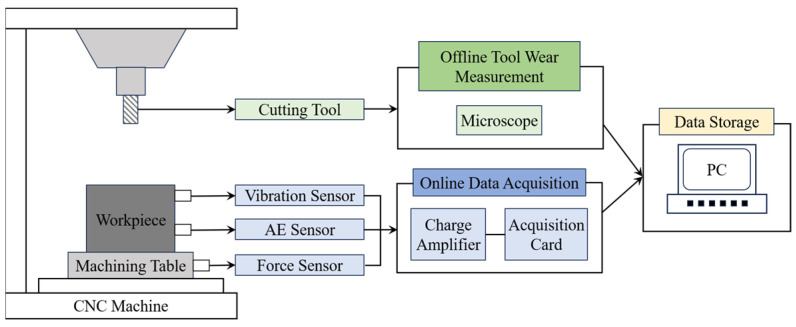
Experimental setup of the PHM2010 dataset.

To objectively evaluate the model performance of different input paradigms, this paper uses the high-speed CNC milling experimental dataset published by the PHM Society in 2010 [64]. Figure 3 displays the schematic diagram of the experimental setup. All milling experiments were conducted on a Roders Tech RFM760 CNC machine. The milling cutter was a ball-tipped carbide cutter, and the workpiece material was HRC52 stainless steel.

Table 1 lists the detailed experimental conditions. In each machining process, the spindle speed was set to be 10,400 r/min; the feed rate in the x-direction was set to be 1555 mm/min; the cutting depth in the y-direction was set to be 0.2 mm; and the cutting depth in the z-direction was set to be 0.125 mm. In order to collect the signals of the tool in real time during the machining process, a Kistler 9265B three-way dynamometer is installed between the table and the workpiece to collect the cutting force signals in x-, y-, and z-directions. Three Kistler 8636C piezoelectric acceleration sensors were mounted on the workpiece to collect vibration signals in three directions. At the same time, a Kistler 8152 acoustic emission sensor mounted on the workpiece was used to collect the high-frequency stress waves generated by the cutting process. As a result, seven channels of analog signals were collected and subsequently converted to digital signals by a DAQ NI PCI1200 acquisition card with a sampling frequency of 50 kHz.

**Figure 4:**
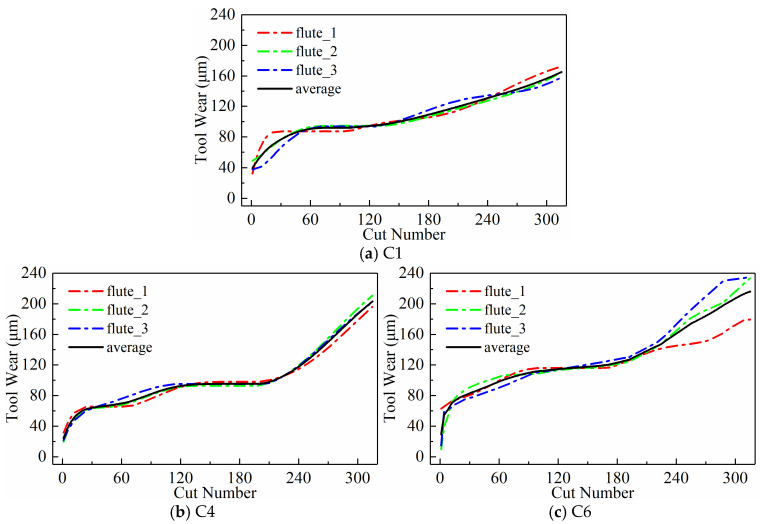
Wear values of the three tools.

Each tool underwent 315 milling operations in total. The Leica MZ12 microscope was used to measure the wear values of the three cutting flutes of the milling tool offline as the tool completed the end milling of 108mm along the x-direction. Although the machining signals of 6 tools were collected during the experiment, only the wear values of tools C1, C4, and C6 were measured. Therefore, the signal data of these three tools were selected as the dataset for this study. According to the recommendation of ISO 8688-2 (1989) [65], the average wear value of the three cutting edges is taken as the actual wear value of the tool, as shown in Figure 4.

### 2.4. Display of Time Series Generated by Different Input Paradigms

**Figure 5:**
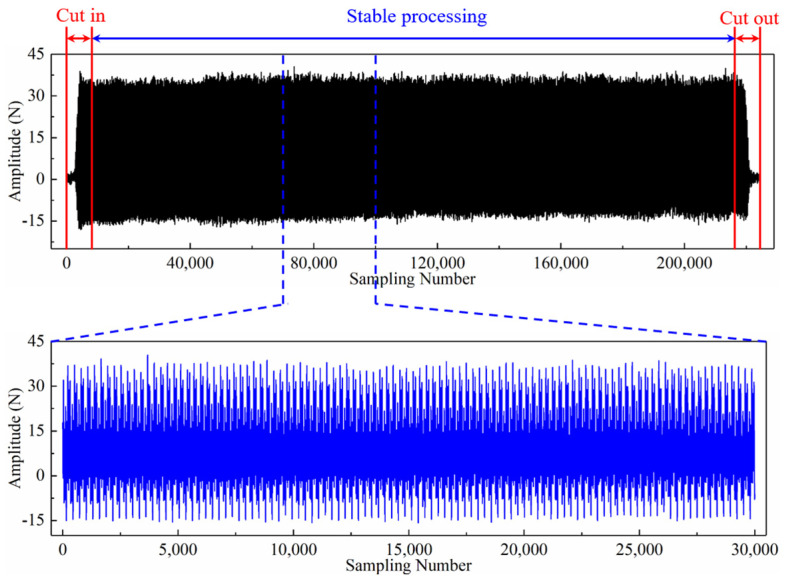
Raw signal of x-direction cutting force collected during the 150th machining process of the tool C6.

Based on the above dataset, different input paradigms are explored in this paper. This section will display the newly generated time series resulting from the application of various input paradigms. As an example, Figure 5 shows the raw signal of the x-direction cutting force that was collected during the 150th machining process of tool C6. Firstly, we show the necessary preprocessing of raw signal data. Each time the tool completes a cutting operation, the collected raw signal data can be divided into three stages: cut in, stable cutting, and cut out. Hence, it is necessary to delete the invalid cut in and cut out data in the sensor data, retaining only the stable data during tool machining. Then, to fairly compare the performance of different input paradigms, a segment of data with a length of 30,000 from the stable machining stage is extracted, as illustrated in the enlarged part at the bottom of Figure 5. The input paradigms are then proposed using the same signal segment.

To ensure a fair comparison, the length of the newly generated time series must also be the same. Studies have pointed out that selecting the length of the signal needs to consider two criteria [53]: (1) The data points must be sufficient to better capture all features of the signal; (2) they should be as short as possible to reduce computation time. The design methods of the different input paradigms were introduced in Section 2.2, compared to the equal interval number $N_{d}$ and the number of subsequences $N_{s}$ which can be arbitrarily set, and the number of periods $N_{p}$ is restricted. To ensure reasonableness, the minimum value of the number of periods is set to $N_{p}=1$. According to the processing parameters listed in Table 1, it can be calculated by Equation (5) that the number of sampling points in one rotation period is approximately 300. Thus, in the performance comparison of different input paradigms, the length of the newly generated time series is set to 300 to achieve a reasonable balance between the two criteria mentioned above.

**Figure 6:**
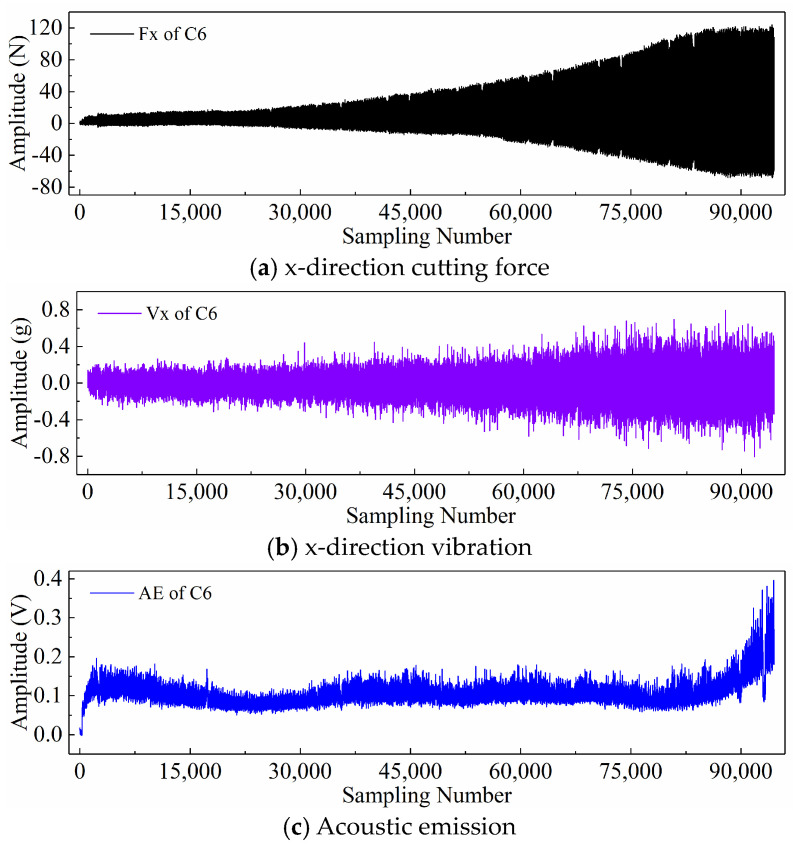
New time series of the tool C6 generated with the downsampling paradigm.

**Figure 7:**
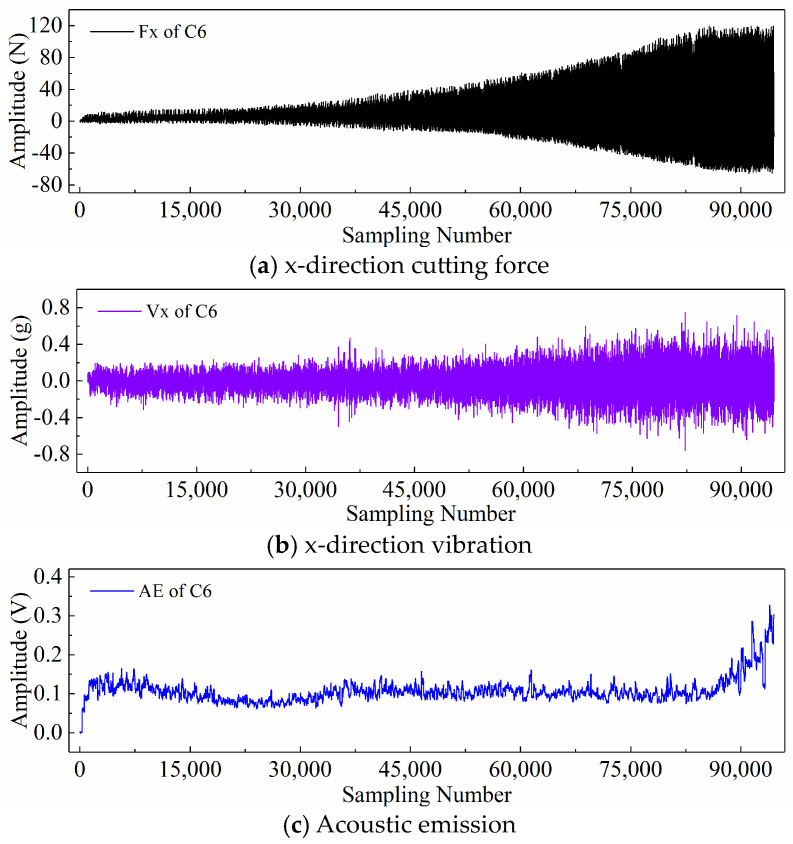
New time series of the tool C6 generated with the periodic paradigm.

**Figure 8:**
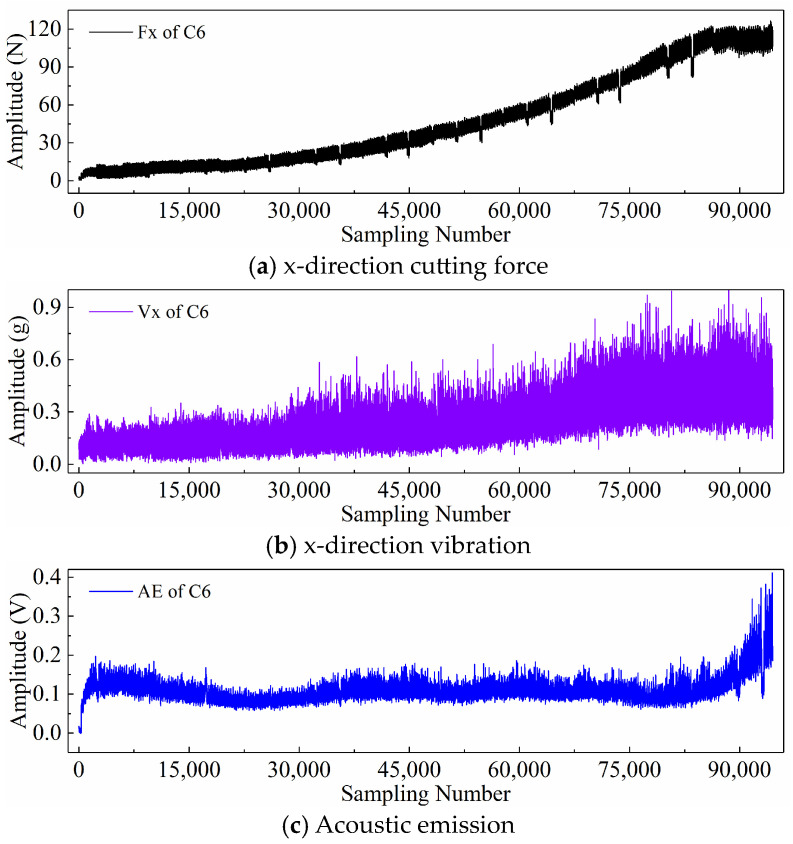
New time series of the tool C6 generated with the subsequence paradigm (where temporal indicator is maximum value).

Finally, taking the x-direction cutting force signal “$F_{x}$”, x-direction vibration signal “$V_{x}$”, and acoustic emission signal “*AE*” during the wear process of tool C6 as examples, Figure 6, Figure 7, and Figure 8 respectively display the newly generated sequences processed by the downsampling paradigm, periodic paradigm, and subsequence paradigm (with the temporal indicator defaulting to the maximum value). Overall, under the three input paradigms, the amplitude of all sensor signals increases with the degree of wear, indicating a favorable correlation between these newly generated time series and the degree of tool wear. Therefore, it is feasible to predict tool wear using the newly generated time series processed by the three input paradigms introduced in this paper. For the same channel sensor signal, the waveforms of the newly generated time series with different input paradigms are not the same. This is especially true for the cutting force and vibration signals, which are thought to be the most sensitive to changes in tool wear. This suggests that the newly generated time series may contain varying tool wear information, which the subsequent sections will further verify through model performance.

## 3. TCM Method Based on an Improved CNN-BiLSTM Hybrid Model

### 3.1. Model Architecture

**Figure 9:**
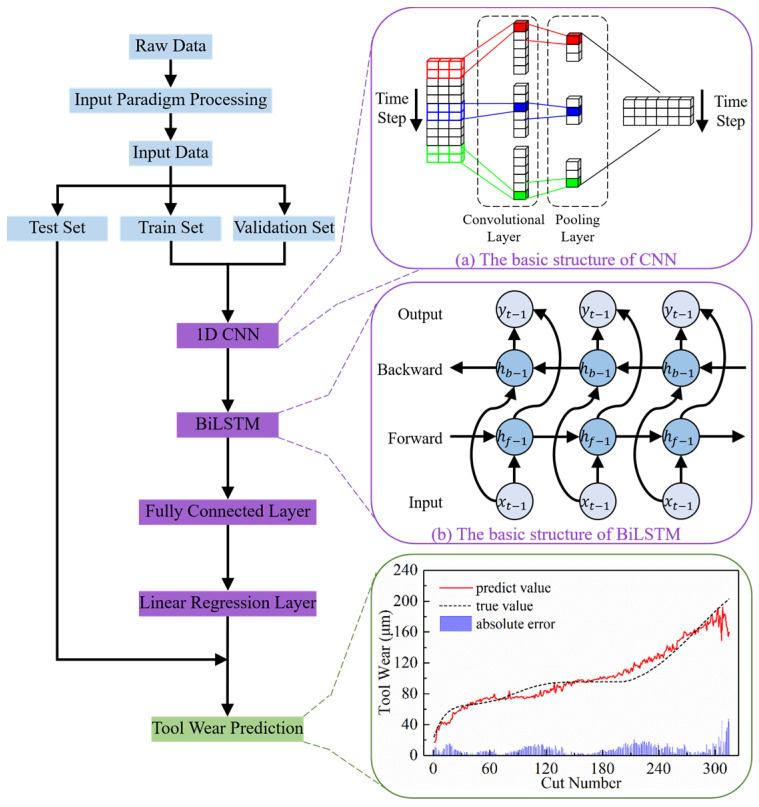
Framework of TCM method based on CNN-BiLSTM.

As mentioned in the Introduction, the latest trend in TCM research is to look into hybrid models that combine CNNs and RNNs. This is because there is a complex spatiotemporal correlation between the multi-channel sensor signal data collected during the tool machining process and tool wear. To this end, this paper designs a CNN-BiLSTM model that combines a one-dimensional CNN (1D CNN) and bidirectional LSTM (BiLSTM), with the corresponding framework shown in Figure 9.

#### 3.1.1. CNN

Figure 9a illustrates the basic structure of the 1D CNN, which primarily consists of convolutional layers and pooling layers to achieve feature extraction and dimensionality reduction [42]. The convolutional layer applies multiple filters to the input time series data, generating feature maps. The pooling layer then compresses each generated feature map to produce important features, effectively extracting spatial information from the multi-channel sensor signals. Suppose that the original signal $x_{i}\in R^{t\times c}$ (where t represents the number of sampling points and c represents the number of signal channels) is input to the CNN layer with alternating convolution and pooling operations. The mathematical expression for the above operation can be seen as [41]:

(6) $$X_{i}^{l}=\sum_{k\in c_{i}}x_{k}^{l-1}\cdot W_{ki}^{l}+b_{i}^{l}$$

(7) $$\mu_{i}^{l}=\sigma (X_{i}^{l})$$

(8) $$P_{i}=\max\;(X_{i}^{l})$$

where $X_{i}^{l}$ represents the *i*th feature map of the *l*th layer, $x_{k}^{l-1}$ represents the *k*th output feature map of the previous layer, $W_{ki}^{l}$ and $b_{i}^{l}$ represents convolution kernel weight matrix and bias, σ represents a nonlinear activation function.

It can be said that the CNN acts as a feature extractor, providing better sequence representation for the subsequent BiLSTM model compared to the complex original input sequences.

#### 3.1.2. BiLSTM

In fact, the tool wear at the current moment is the result of the gradual accumulation of wear during past cutting processes and will directly affect the wear trend of subsequent cutting processes. This imposes higher requirements on the predictive model’s ability to capture the time-dependent characteristics of the signals [41]. LSTM effectively addresses the gradient problem in RNNs by introducing forget gates, input gates, and output gates [66], thus capturing the long-term dependencies of sensor signal data. The forget gate $f_{t}$ controls the retention and forgetting of information, the input gate $i_{t}$ decides which of the current input information $x_{t}$ and the output $h_{t-1}$ from the previous LSTM unit will be kept, and the output gate $o_{t}$ calculates the output $h_{t}$ of the LSTM unit by multiplying the cell state with tanh. The mathematical equations for the three gates are as shown below [67]:

(9) $$f_{t}=\sigma (W_{f}\cdot \left[h_{t-1},x_{t}\right]+b_{f})$$

(10) $$i_{t}=\sigma (W_{i}\cdot \left[h_{t-1},x_{t}\right]+b_{i})$$

(11) $${\tilde{C}}_{t}=tanh(W_{C}\cdot \left[h_{t-1},x_{t}\right]+b_{C})$$

(12) $$C_{t}=f_{t}*C_{t-1}+i_{t}*{\tilde{C}}_{t}$$

(13) $$o_{t}=\sigma (W_{o}\cdot \left[h_{t-1},x_{t}\right]+b_{o})$$

(14) $$h_{t}=o_{t}*\tanh\;(C_{t})$$

where *W* is the weight matrix, b is the bias term, σ is the activation function, $C_{t}$ is the cell state, ∗ denotes a multiplication of vector elements, ${\tilde{C}}_{t}$ is the new candidate value vector, $h_{t}$ is the output of the LSTM unit.

BiLSTM, a variant of LSTM, adopts a bidirectional structure with forward LSTM and backward LSTM, ensuring dual dependencies from the past to the future and reverse dependencies. Among them, take ${\vec{h}}_{t}\;and\;{\overset{\leftarrow }{h}}_{t}$ as the outputs of the forward LSTM and backward LSTM:

(15) $${\vec{h}}_{t}=\vec{LSTM}(h_{t-1},x_{t},C_{t-1})$$

(16) $${\overset{\leftarrow }{h}}_{t}=\overset{\leftarrow }{LSTM}(h_{t+1},x_{t},C_{t+1})$$

Then, the output $y_{t}$ of BiLSTM, after the forward and backward calculations, can be expressed as:

(17) $$y_{t}={\vec{h}}_{t}\;⨁\;{\overset{\leftarrow }{h}}_{t}$$

Recent years have demonstrated its ability to capture dependencies from both the past and future, leading to more effective monitoring of tool wear trends [35]. The basic architecture of BiLSTM, as depicted in Figure 9b, mirrors that of unidirectional LSTM, with the only difference being the information flow within the layers [37].

### 3.2. Model Training and Testing

Figure 9 also shows the flow of the TCM method proposed in this paper, described in detail as follows:

#### 3.2.1. Data Preprocessing

Before directly inputting the newly generated time series from the previous section into the DL model, necessary preprocessing, including normalization and dataset partitioning, is required. Figure 6, Figure 7 and Figure 8 demonstrate that even after processing the original signal data with different input paradigms, the magnitudes of the new time series in different channels remain different. To avoid model internal weights biasing towards higher-magnitude features, the Z-score normalization method is used to convert the input data to the same magnitude. The calculation formula is as follows:

(18) $$z_{i}=\frac{x_{i}-\mu_{i}}{\sigma_{i}}$$

where $x_{i}$ represents the input data and $\mu_{i}$ and $\sigma_{i}$ represent the mean and variance, respectively.

Since there are only three tool datasets (C1, C4, and C6) with limited size, the threefold cross-validation method is used to verify the model’s generalization ability and avoid overfitting. Specifically, any two of the three tool datasets are divided into training and validation sets in a ratio of 8:2; the remaining tool dataset is used as the test set. Finally, the model’s performance is evaluated based on the three test results.

#### 3.2.2. Implementation Details

This paper designs the CNN-BiLSTM model, a hybrid “end-to-end” model that can directly use the new time series data obtained in Section 2 as input, eliminating the need for cumbersome feature engineering and obscure expertise. First, the convolution algorithm of a single-layer CNN is used to extract the spatial features of the multi-sensor raw signal data. These high-dimensional features have been shown to have greater potential in reflecting tool wear [67]. Here, the single-layer CNN is improved by adding batch normalization (BN) operations after the convolutional layer and before the activation function, restoring the data to a standard normal distribution. This eliminates the need for the convolutional layer parameters to frequently adapt to constantly changing distributions, thereby accelerating the model training process. Next, a stack of BiLSTM with 4 layers and 64 hidden units is built on top of the single-layer CNN. The abstract features obtained by the CNN are input into the first BiLSTM layer, with the hidden states of the LSTM units connected through forward and backward transmission to form the input of the second BiLSTM layer, and the same propagation process is repeated. In this way, through forward and backward bidirectional operations, the learning of sequence features based on known time series and reverse position sequences is enhanced, thereby achieving bidirectional long-term temporal dependencies for the tool wear sequence. Finally, a nonlinear regression model containing two fully connected layers and a linear regression layer is designed on top of the BiLSTM to map the spatiotemporal features of the multi-sensor signal data learned by the CNN-BiLSTM, thereby predicting the corresponding tool wear values.

To better train the above-designed composite model, the training parameters were fine-tuned empirically. The main objective during model training is to minimize the loss function, and this paper uses mean square error (MSE) as the loss function. The parameters are adjusted through backpropagation using the Adam optimizer to minimize the loss. During the model training phase, it is particularly noteworthy that the input data, obtained after processing with the input paradigm, significantly reduce the data scale compared to the raw signal data. Additionally, the newly generated sequence data must sequentially pass through the CNN layer, BiLSTM layer, and fully connected layer. As the number of network layers increases, these factors make overfitting a serious issue. In addition, for threefold cross-validation, there is a significant difference in the data distributions of C1, C4, and C6 [56], which our previous work shows further increases the risk of overfitting. Therefore, dropout and early stopping techniques were introduced to suppress the overfitting problem of the CNN-BiLSTM hybrid model and further enhance its predictive performance. Dropout randomly removes hidden neurons at a set rate. This means that these neurons do not take part in the model’s forward propagation, which makes the model less reliant on local features and better at generalization [41]. This paper sets the dropout rate at 0.2. In the case of early stopping, when the model’s loss on the validation set no longer decreases after a certain amount (patience value) of continuous iterative training, training stops. This determines the minimum number of iterations needed to train the model, minimizing the possibility of overfitting [35]. This paper sets the patience value at 20. Table 2 lists the specific settings of the primary hyperparameters. These hyperparameters are tuned based on the model’s performance on the validation dataset, and the value of the hyperparameter with the lowest loss value is chosen.

Finally, the performance of the well-trained composite model is evaluated using the test set. This paper employs root mean square error (RMSE) and mean absolute error (MAE), which are commonly used in regression prediction, as evaluation metrics. RMSE is very sensitive to extreme outliers, while MAE is relatively robust and can better reflect the actual situation of identification errors. The smaller the MAE and RMSE values, the better the model’s overall performance. The calculation formulas for these two metrics are as follows:

(19) $$MAE=\frac{1}{n}\sum_{i=1}^{n}\left|{\bar{y}}_{i}-y_{i}\right|$$

(20) $$RMSE=\sqrt{\frac{1}{n}\sum_{i=1}^{n}{\left({\bar{y}}_{i}-y_{i}\right)}^{2}}$$

where n is the number of wear values, $y_{i}$ is the ith actual wear value, and ${\bar{y}}_{i}$ is the ith predicted wear value.

The proposed model is build based on Pytorch 2.1.2 framework with CUDA 12.1 and Python 3.9. The computer configuration is Intel(R) Core(TM) i5-9400F CPU, 16 GB of RAM, NVIDIA GeForce GTX 1660Ti, and Windows10 Professional system.

## 4. Results and Discussion

### 4.1. Exploration of Different Input Paradigms

#### 4.1.1. Model Performance of Different Input Paradigms

**Figure 10:**
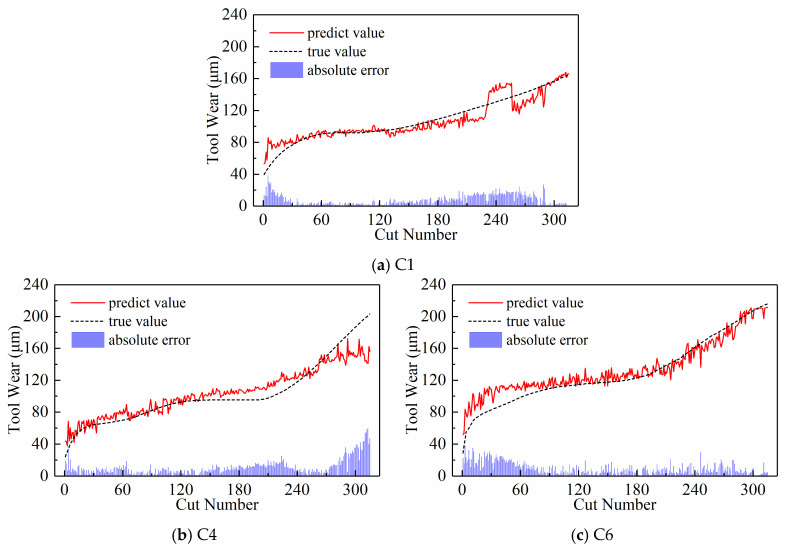
Prediction results of the downsampling paradigm.

**Figure 11:**
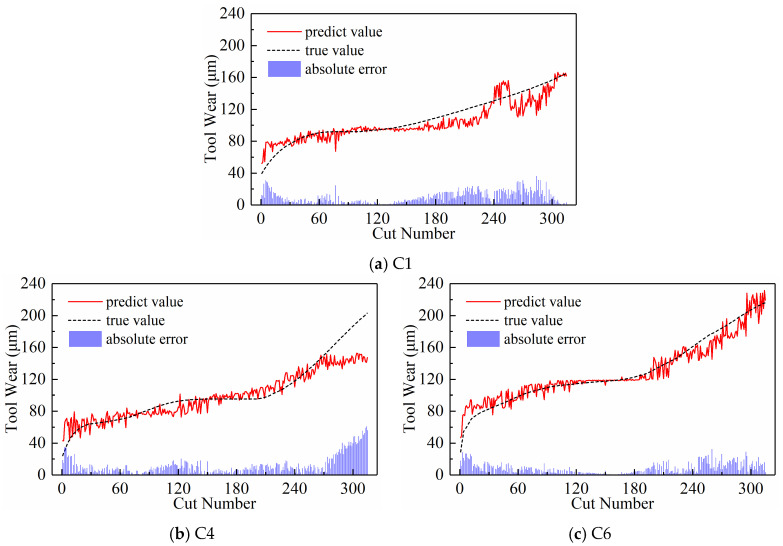
Prediction results of the periodic paradigm.

**Figure 12:**
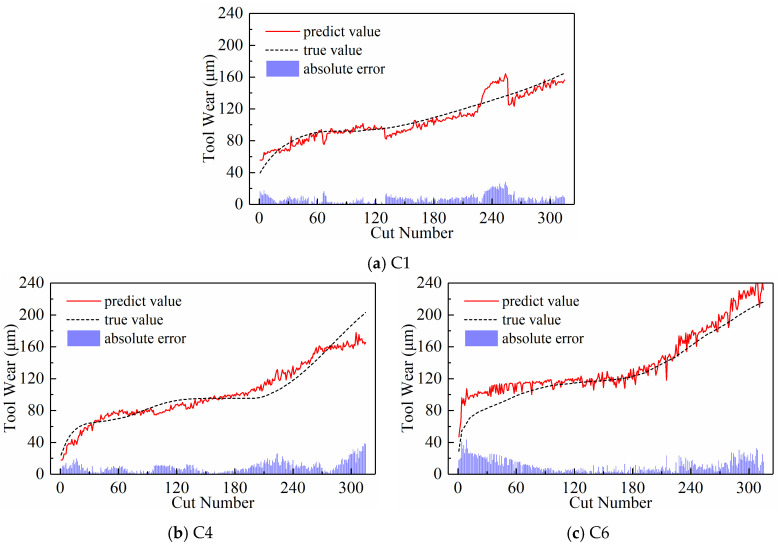
Prediction results of the subsequence paradigm.

We analyzed the predictive performance of the CNN-BiLSTM model using the new time series generated by the three input paradigms shown in Section 2.3. Note that the newly generated sequences here have a length of 300. The predictive results of the three input paradigms are shown in Figure 10, Figure 11, and Figure 12, respectively, where the red solid line represents the predicted value of tool wear, the black dashed line represents the actual value of tool wear, and the bottom histogram represents the absolute error between the predicted value and the actual value. It is well known that the tool wear process can be divided into three stages: initial wear, normal wear, and severe wear. Overall, the tool wear prediction curves of the three input paradigms to some extent follow the actual wear trend and basically capture the three stages of tool wear. This indicates that the three paradigms designed in this paper are effective in predicting tool wear. In order to further quantitatively analyze the predictive performance of the model, the detailed data of the evaluation indicators are listed in Table 3 below.

Table 3 lists the model’s MAE and RMSE values for all test sets under the three input paradigms. The table reveals that the subsequence paradigm has achieved nearly optimal performance, with the exception of tool C6, where the periodic paradigm yields the best results. Overall, compared to the downsampling paradigm and the periodic paradigm, using the input data obtained from the subsequence paradigm for model training has improved the model’s overall performance to some extent. In addition, Table 3 also lists the computational time of the model under the three input paradigms. The subsequence paradigm exhibits the highest computational efficiency, with its computational time only 82.45% of that of the downsampling paradigm and 71.12% of that of the periodic paradigm.

#### 4.1.2. Dimensionality Reduction Potential of Different Input Paradigms

The comparison in the previous section was based on the same input sequence length (300), thus providing only a preliminary conclusion. As stated in the Introduction, one of the starting points of this paper is to investigate how to minimize the scale of input data while ensuring predictive performance. Therefore, it is necessary to explore the performance of different input paradigms when the input sequence length changes, in order to clarify their potential for reducing dimensionality. Obviously, with the decrease in input sequence length, if the model’s performance improves, it indicates that the input paradigm’s dimensionality reduction potential is high. Therefore, this paper selects different sequence lengths of 30, 150, 300, and 1500 and accordingly adjusts the parameters of the three input paradigms as follows:

- Downsampling paradigm: Set intervals Nd to 1000, 200, 100, and 20, respectively.
- Periodic paradigm: Set the number of cycles Np to 0.1, 0.5, 1, and 5, respectively.
- Subsequence paradigm: Set the number of subsequences Ns to 30, 150, 300, and 1500, respectively.

**Figure 13:**
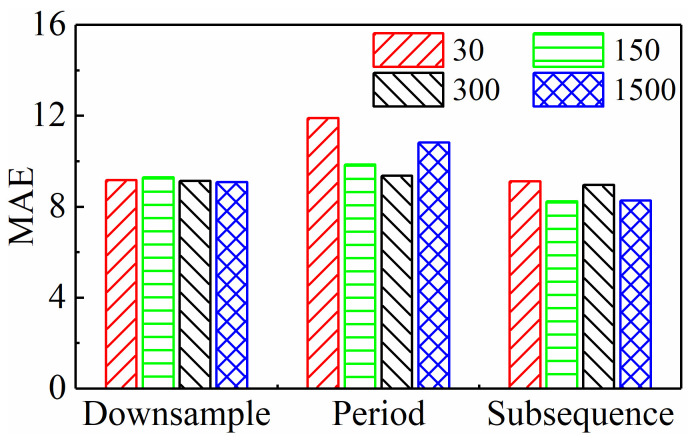
Average MAE of three input paradigms with different input sequence lengths.

**Figure 14:**
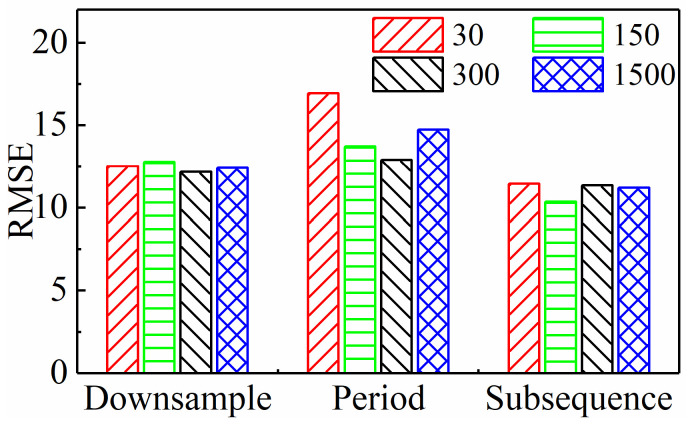
Average RMSE of three input paradigms with different input sequence lengths.

Under all of the above conditions, Table 4 lists the model’s MAE and RMSE on three test sets. To compare the prediction performance more intuitively, the average values of these two indicators under each input paradigm were calculated, as shown in Figure 13 and Figure 14, respectively. For the downsampling paradigm, the model’s MAE and RMSE do not change much as the sequence length goes down. This means that the model’s performance does not change much as the sequence length goes down. This might be because the original data had a high sampling frequency. For the periodic paradigm, as the sequence length decreases, the time series features contained within the sequence are reduced and even no longer reflect a complete period, resulting in a deterioration of the model performance. Thus, for any input sequence length, its MAE and RMSE are the largest among these three paradigms. However, for the subsequence paradigm, its MAE and RMSE are lower than those of the other two paradigms, which is mainly attributed to the time series generated by the subsequence paradigm that better maintains the integrity of the original sequence and thereby more effectively retains the feature information of tool wear contained in the original data.

**Figure 15:**
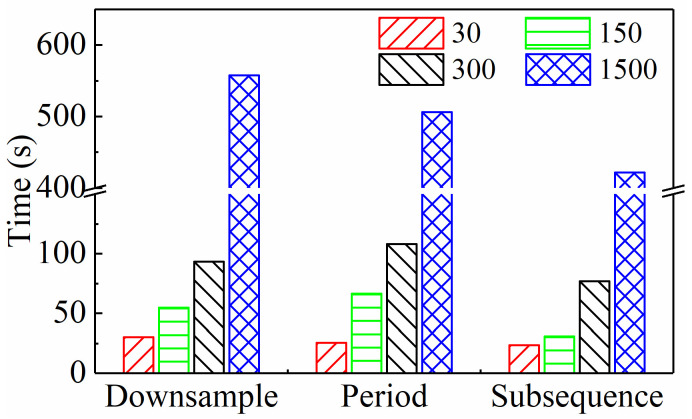
Computation time of three input paradigms with different input sequence lengths.

In addition, Figure 15 shows the computation time under all the above conditions. It is clear from the figure that as the input sequence length decreases, the model’s computation time significantly decreases, and the computational efficiency significantly improves. This paper’s data dimensionality reduction study plays a crucial role in achieving real-time prediction and industrial deployment. Furthermore, the figure clearly shows that for any input sequence length, the subsequence input paradigm has a much shorter running time than the other two paradigms, confirming that it has the highest computational efficiency.

In summary, compared to the downsampling paradigm and the periodic paradigm, the subsequence paradigm can predict tool wear more accurately and quickly under further reduced sequence lengths. Considering both accuracy and efficiency, the subsequence paradigm has the greatest potential for dimensionality reduction among the three input paradigms designed in this paper. It offers significant advantages in terms of improving TCM’s real-time performance and effectiveness.

### 4.2. Further Exploration of the Subsequence Paradigm

The previous section’s preliminary discussion clarified the rationale and superiority of the subsequence paradigm compared to the downsampling paradigm and the periodic paradigm. This section goes into more detail about the subsequence paradigm because the number of subsequences and temporal indicators in it directly affect the data content of the newly created time series and can also have a direct effect on how well the model works in the subsequence paradigm. It evaluates the impact of the number of subsequences and temporal indicators on model performance to further enhance the model performance of the subsequence paradigm.

#### 4.2.1. The Impact of Different Numbers of Subsequences on Model Performance

To clarify the impact of the number of subsequences on model performance, this subsection analyzes in detail the changes in model performance as the number of subsequences ranges from 30 to 300 (in intervals of 30).

(1) Display of Newly Generated Time Series

**Figure 16:**
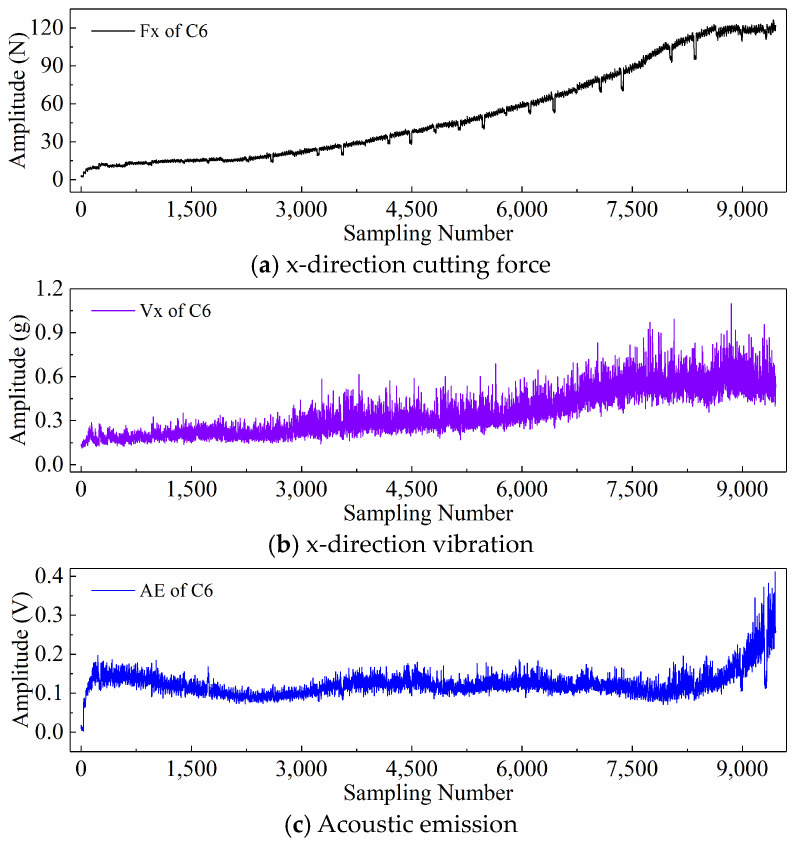
New time series of the tool C6 generated with a subsequence number of 30.

**Figure 17:**
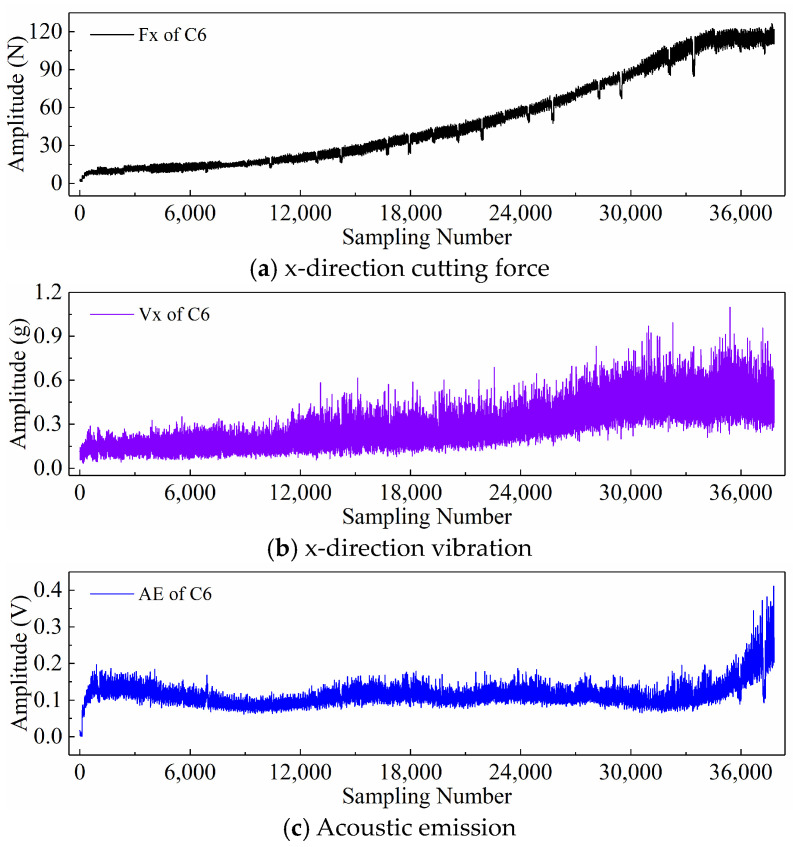
New time series of the tool C6 generated with a subsequence number of 120.

**Figure 18:**
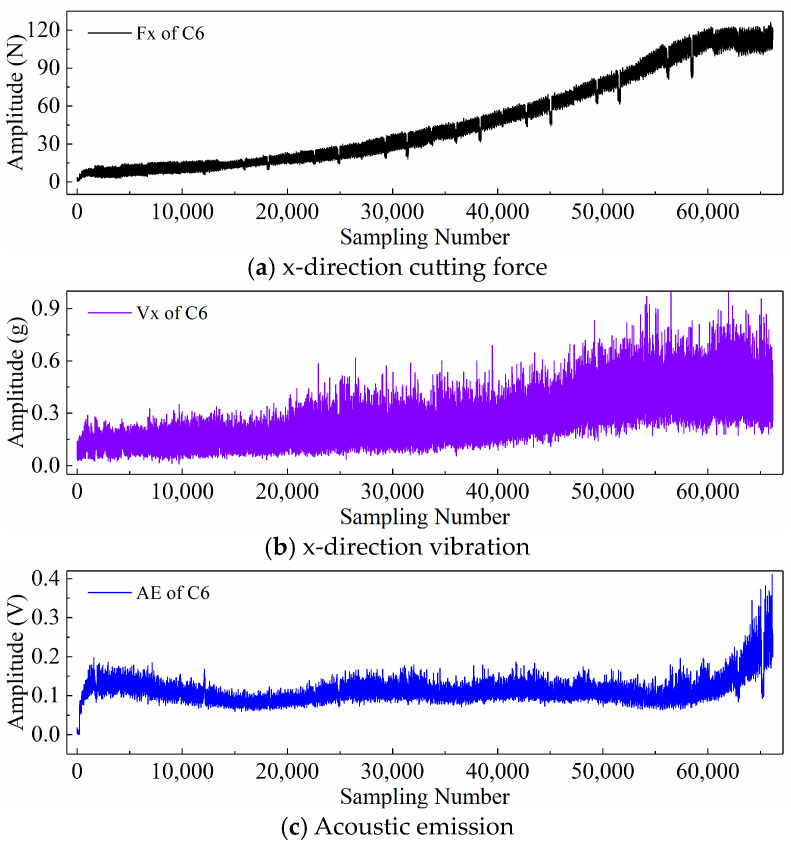
New time series of the tool C6 generated with a subsequence number of 210.

**Figure 19:**
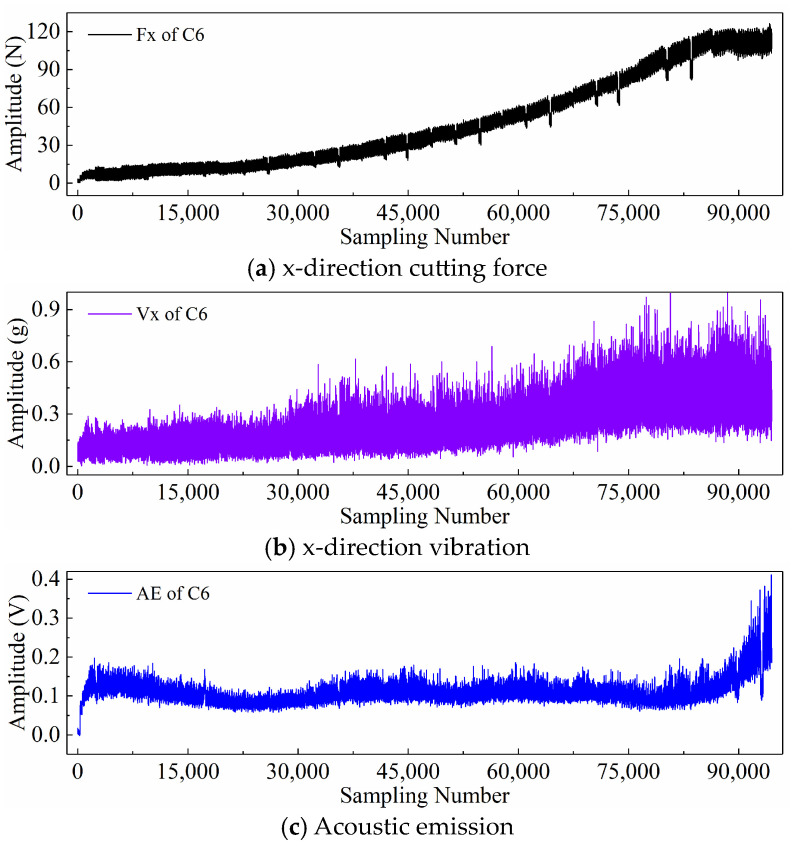
New time series of the tool C6 generated with a subsequence number of 300.

Figure 16, Figure 17, Figure 18, and Figure 19, respectively, display only the newly generated time series with subsequence numbers of 30, 120, 210, and 300 due to space limitations. Visually, as the number of subsequences changes, the waveforms of the newly generated time series based on the same channel sensor signal show similar trends with the degree of tool wear. The most apparent difference is the length of the time series. Different lengths may lead to differences in the tool wear information contained in the time series. Further discussion will be conducted in conjunction with the model performance below.

(2) Predictive Performance

**Figure 20:**
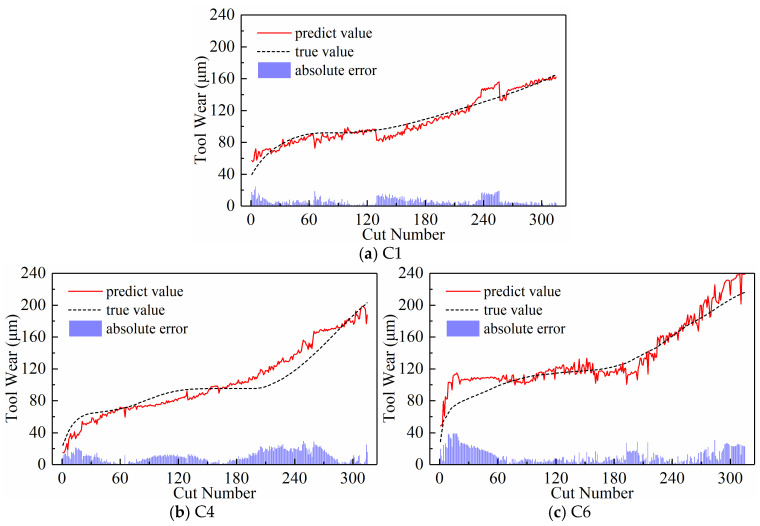
Prediction results of the subsequence paradigm with a subsequence number of 30.

**Figure 21:**
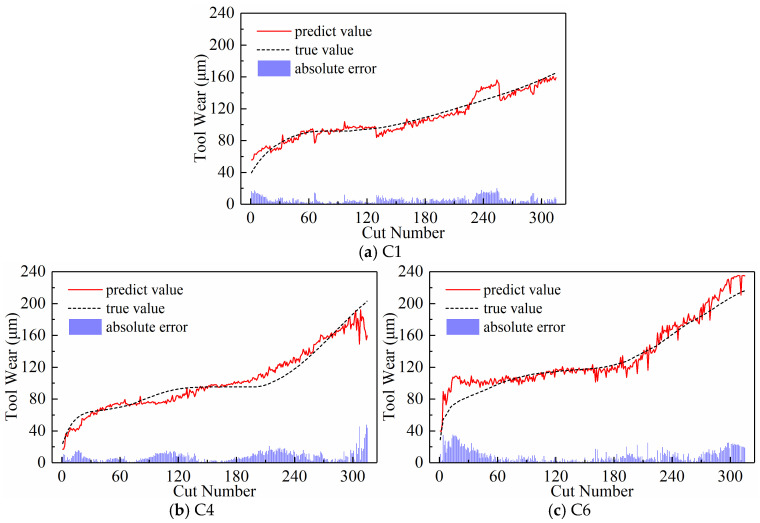
Prediction results of the subsequence paradigm with a subsequence number of 120.

**Figure 22:**
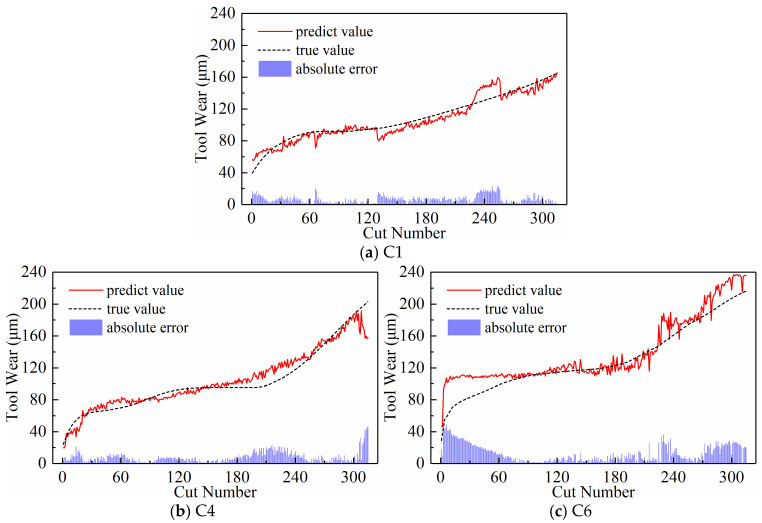
Prediction results of the subsequence paradigm with a subsequence number of 210.

**Figure 23:**
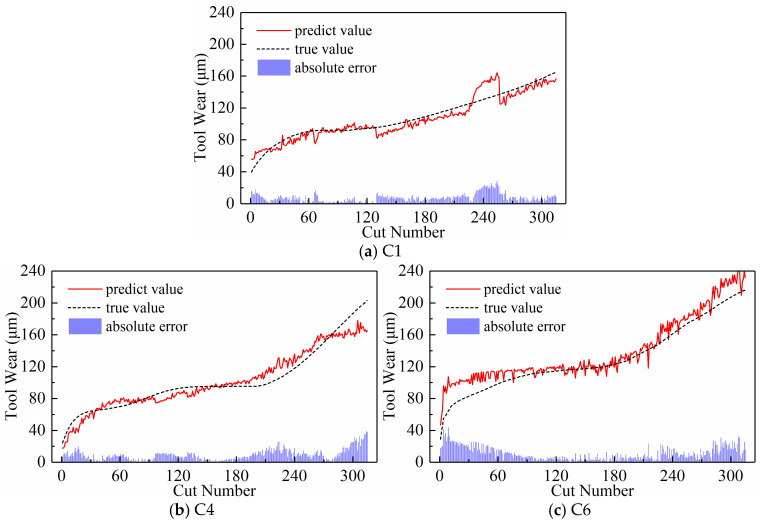
Prediction results of the subsequence paradigm with a subsequence number of 300.

Figure 20, Figure 21, Figure 22, and Figure 23 show the prediction curves of the model on three test sets with subsequence numbers of 30, 120, 210, and 300, respectively. The figures demonstrate a strong match between the tool wear prediction curves and the actual wear curves, effectively capturing the overall tool wear trend and clearly distinguishing between the three distinct wear stages. However, it is also evident that there are noticeable errors between the predicted and actual values of tool wear in the local details, especially in the early and late stages of wear of the C6 tool. The fast wear rate of the tool primarily contributes to this, as it offers fewer features for the model to learn.

**Figure 24:**
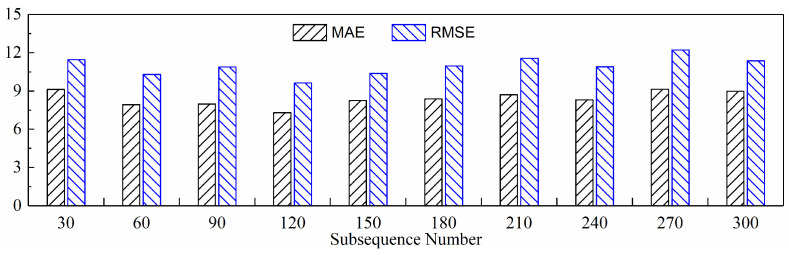
Average predictive performance with different subsequence numbers.

Table 5 lists the evaluation metrics of the model’s prediction performance on the three test sets for the entire number of subsequences. Similarly, the average values of these evaluation metrics were calculated, as shown in Figure 24, to facilitate a more intuitive comparison. It can be observed that when the number of subsequences input to the model is too small, both the MAE and RMSE of the model increase, resulting in poorer prediction performance. This is because the subsequence length is too long, causing more sequence data to be lost during the data extraction process, making it difficult for the newly generated sequence to maintain the overall sequence characteristics of the original sequence. Consequently, the model struggles to learn the complete tool wear information. However, when the number of subsequences is too large, the length of each subsequence is too short, resulting in each subsequence containing too little information to acquire sufficient local feature information, and the model’s prediction performance similarly deteriorates, with both MAE and RMSE increasing. Additionally, an excessive number of subsequences leads to an excessively long input sequence, complicating the model’s calculation process and significantly increasing the training time. Based on the cross-validation of MAE and RMSE in Table 5 and Figure 24, it can be concluded that the model’s prediction performance is optimal when the number of subsequences is set to 120.

#### 4.2.2. Impact of Different Temporal Indicators on Model Performance

As is well known, the maximum value, minimum value, and mean value are the most commonly used temporal indicators. This section discusses in detail the changes in model performance when using these three common temporal indicators to clarify the impact of different temporal indicators on model performance.

(1) Display of Newly Generated Time Series

**Figure 25:**
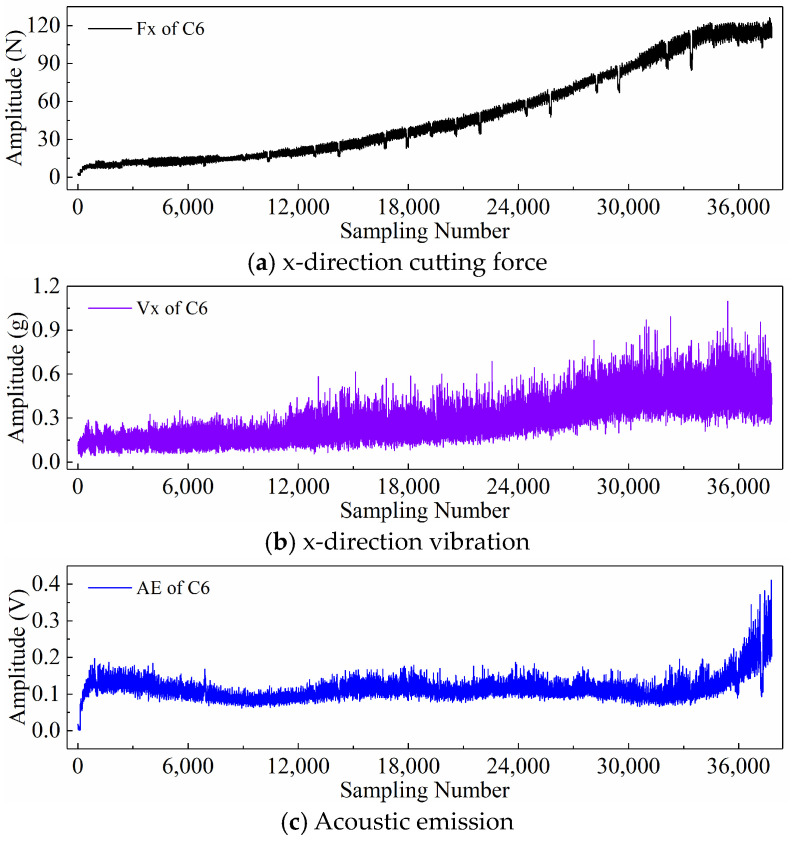
New time series of the tool C6 generated with the maximum value as the temporal indicator.

**Figure 26:**
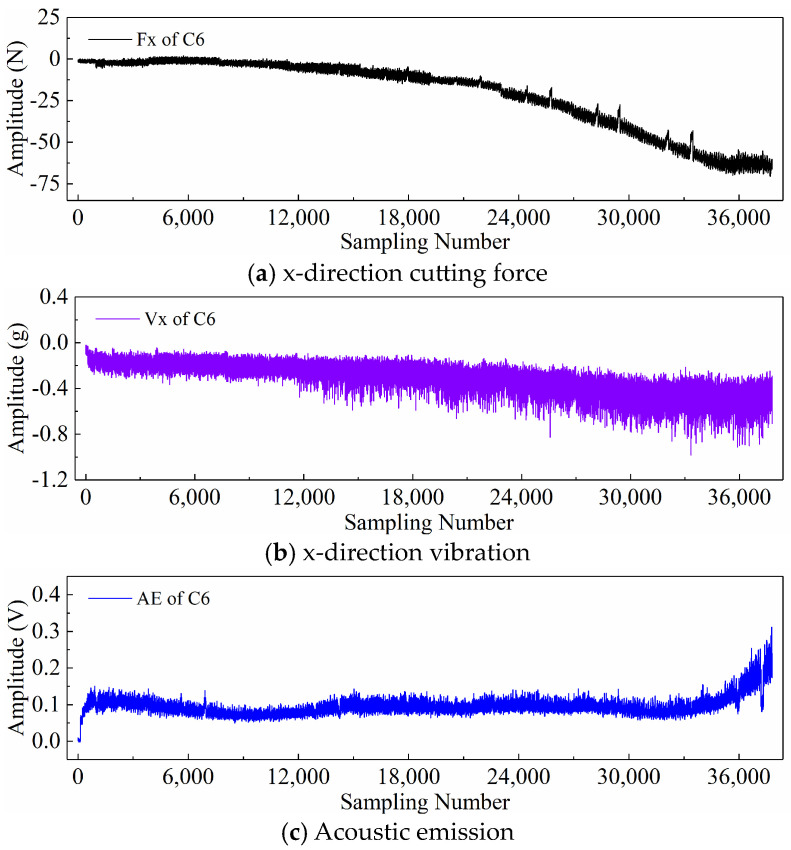
New time series of the tool C6 generated with the minimum value as the temporal indicator.

**Figure 27:**
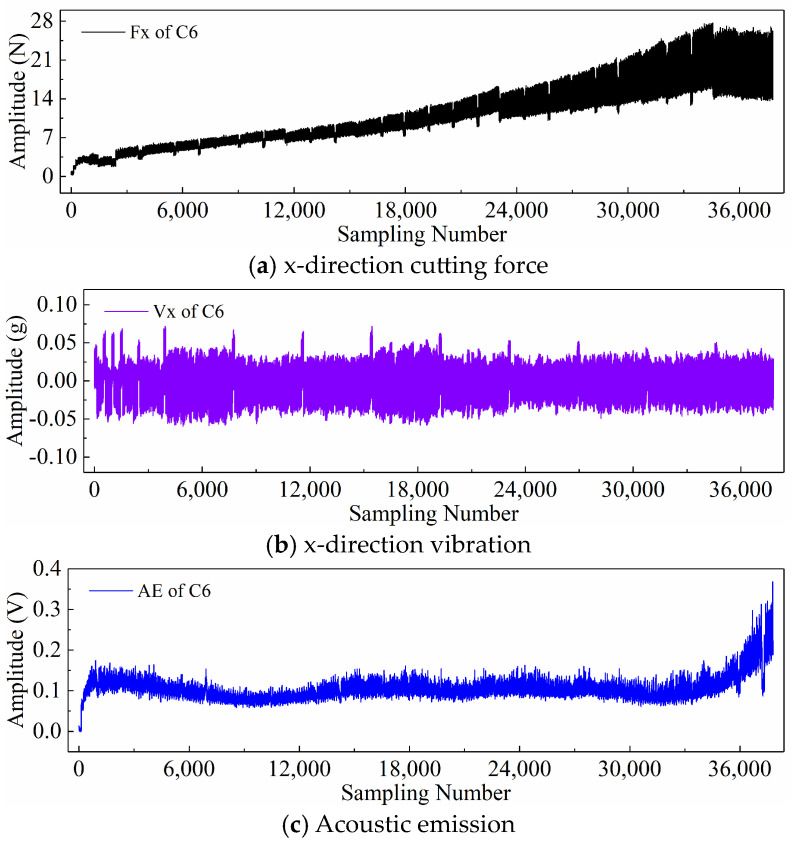
New time series of the tool C6 generated with the mean value as the temporal indicator.

Figure 25, Figure 26, and Figure 27 show the newly generated sequences when the temporal indicators are the maximum value, minimum value, and mean value, respectively. Using different temporal indicators, the waveform of the new sequences generated from the original acoustic emission signals shows a similar trend with the degree of tool wear, as shown in the figures. However, the waveforms generated by the new sequences based on the original cutting force and vibration signals show significant differences. In particular, the new sequences of cutting force and vibration signals made with the highest value as the time indicator are linked to the trend of tool wear in a positive way, while the new sequences made with the lowest value as the time indicator are linked to it in a negative way. The new sequence of cutting force signals exhibits a positive correlation with the tool wear trend when the mean value serves as the temporal indicator, while the amplitude of the new vibration signals does not significantly alter with the degree of tool wear. The complex changes in the waveforms of the above new sequences suggest a significant difference in the tool wear information they contain, which will be confirmed in the subsequent model performance comparisons.

(2) Predictive Performance

**Figure 28:**
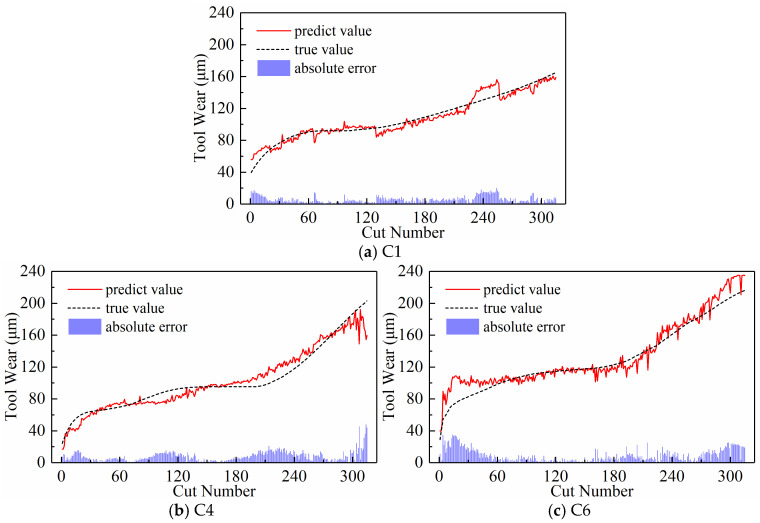
Prediction results of the subsequence paradigm with maximum values as the temporal indicator.

**Figure 29:**
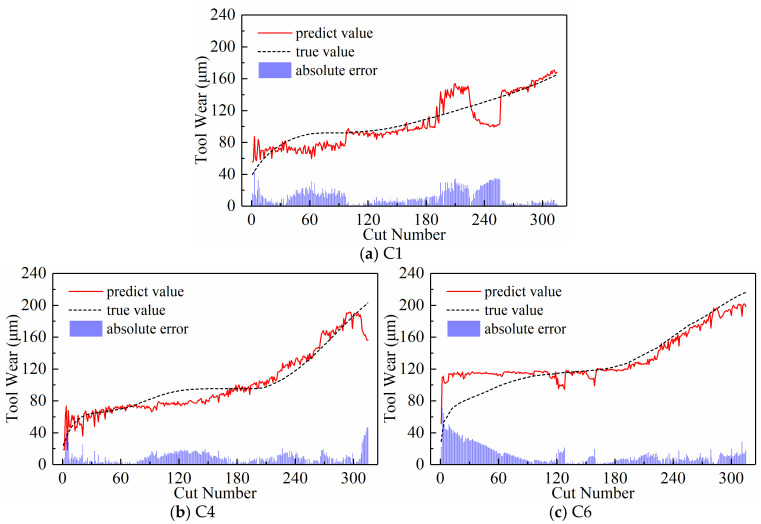
Prediction results of the subsequence paradigm with minimum values as the temporal indicator.

**Figure 30:**
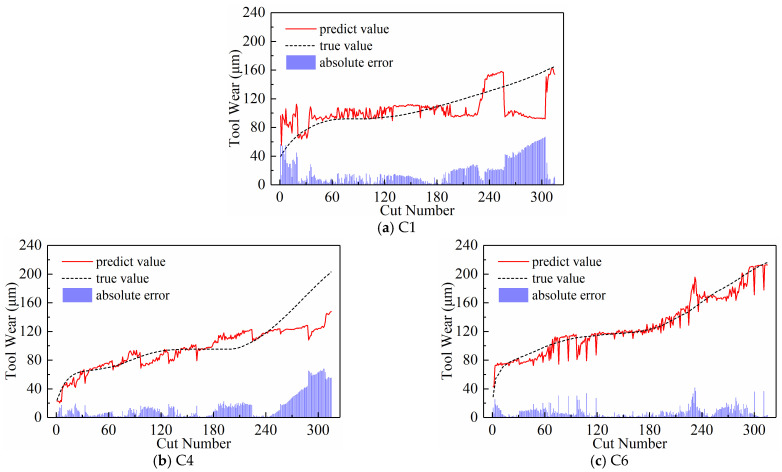
Prediction results of the subsequence paradigm with mean values as the temporal indicator.

The prediction results are presented in Figure 28, Figure 29, and Figure 30, respectively, when the temporal indicator is set as maximum, minimum, and mean values. The figures reveal significant differences in the model’s predicted tool wear trends under the three time-domain indices. Setting the temporal indicator to the maximum value brings the wear prediction curves closer to the actual value curves, with the exception of tool C6. The tool wear prediction results at the maximum value are more closely aligned and stable with the actual tool wear. However, when we set the temporal indicator as the mean value for tool C6, the model’s tool wear prediction appears to more closely align with the actual wear trend.

**Figure 31:**
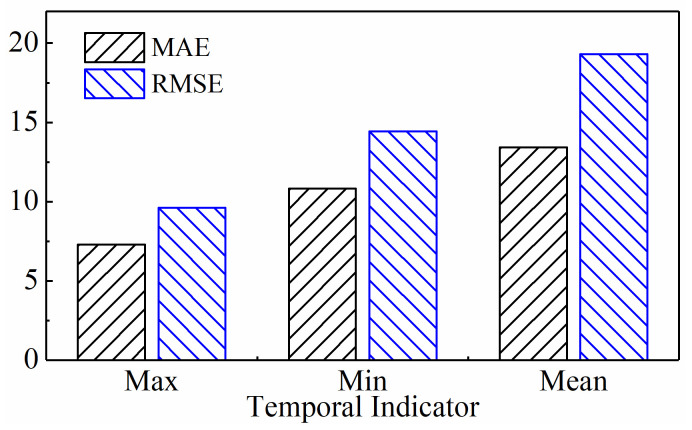
Average predictive performance with different temporal indicators.

Table 6 lists the prediction evaluation metrics of the model on three test sets, including MAE and RMSE, when the temporal indicators are set as maximum, minimum, and mean values. The model for predicting tool wear, using tools C1 and C4 as test sets, shows lower MAE and RMSE when using the maximum value as the temporal indicator compared to the other two temporal indicators. However, when using tool C6 as the test data, both MAE and RMSE only rank second, with the best results achieved by using the mean value as the temporal indicator, as intuitively shown in Figure 30c. The average values of MAE and RMSE in Figure 31 also show that when the temporal indicator is set to the highest value, the model’s MAE is 32.51% lower than the minimum value and 45.67% lower than the mean value. Similarly, when the maximum value is set, the RMSE is 33.32% lower than the minimum value and 50.16% lower than the maximum value. This makes the error rate much lower. Therefore, setting the temporal indicator to the maximum value optimizes the model’s overall prediction performance, confirming the effectiveness and superiority of using the maximum value as the temporal indicator.

An interesting observation to note here is that although the overall performance of the model when the temporal indicator is set as maximum values is better than the minimum and mean values, the model has the smallest MAE and RMSE when the temporal indicator is set as mean value using C6 tools as the test data. This provides an important inspiration that “fusing” the new time series generated from multiple temporal indicators could improve the generalization of the model to different tools. This is just what we are currently working on.

### 4.3. Comparison with Other Methods

The detailed analysis above reveals that the improved CNN-BiLSTM model in this paper achieves optimal prediction performance by selecting the input paradigm as the subsequence paradigm, setting the number of subsequences to 120, and using the maximum value as the temporal indicators. Table 7 confirms the superiority of the proposed method in three aspects by comparing its prediction performance with several classical and contemporary methods using the same dataset.

Firstly, the proposed method uses raw signal data as input, and the MAE and RMSE on the three test sets are significantly smaller than those of traditional ML methods that use manually extracted features as input. This indicates that the proposed method can simplify the modeling process and improve the real-time prediction process without sacrificing prediction performance.

Secondly, this paper designs a CNN-BiLSTM composite model that combines the advantages of CNN and BiLSTM, as detailed in Section 3.1, enabling it to fully learn the spatiotemporal features contained in multi-sensor signals. Therefore, its prediction performance is much better than that of single-architecture models such as CNN, LSTM, and BiLSTM.

Finally, the paper selects the subsequence paradigm from three input paradigms and carefully designs the number of subsequences and temporal indicators. The proposed method outperforms other methods using similar composite models, such as CNN-LSTM and CABLSTM, as the generated new sequence achieves optimal model performance, with the MAE and RMSE of the three test sets being lower.

## 5. Conclusions and Future Works

This paper presents a groundbreaking investigation into the processing techniques for transforming multi-sensor raw data into DL input data, specifically input paradigms. This process ensures a reduction in data scale and a sufficient temporal interpretation of tool wear, enabling the full utilization of the “end-to-end” benefits of DL models in the TCM field. The following are the main conclusions drawn from this exploration:

(1) A new end-to-end framework for tool wear prediction was designed. Firstly, a suitable input paradigm was selected to generate new time series data directly into the model, eliminating the need for complex manual feature extraction. Then an improved CNN-BiLSTM hybrid model was utilized for prediction, capable of capturing the complex spatiotemporal correlation between the multi-sensor data and tool wear.
(2) The subsequence paradigm had the lowest overall MAE and RMSE prediction performance metrics and the shortest computation time compared to the downsampling paradigm and the periodic paradigm. This shows that the subsequence paradigm is a great way to make TCM more effective and faster.
(3) Further in-depth exploration of the subsequence paradigm revealed that the model’s MAE and RMSE were lowest when there were 120 subsequences and the temporal indicator was set to its highest value. This was after threefold cross-validation.
(4) Finally, we demonstrated the superiority of the proposed method by ditching feature engineering, overcoming the limitations of a single model architecture, and constructing high-quality input data by comparing the prediction performance of several classical and contemporary methods using the same dataset.

In conclusion, the exploration of the input paradigm in this paper provides new ideas for achieving end-to-end tool wear prediction. Although the model performs well on the PHM2010 dataset, its ability to generalize to different tools, materials, or machining conditions requires further validation, which may require adjustments to the input data and model configuration. As a follow-up, efforts are underway to enhance the model’s generalization to various tools through the “fusion” of multiple temporal indicators, and we are considering transfer learning in the future to monitor tool wear under diverse operating conditions.

## Figures and Tables

**Table 1:** Milling parameters and experimental equipment.

Milling Parameters	Value	Equipment Type	Equipment Model
Milling method	Climb milling	Machine	Roders RFM760 (Roders, Soltau, Germany)
Cooling method	Dry cutting	Tool	Ball-end carbide milling cutter (Roders, Soltau, Germany)
Spindle speed (r/min)	10,400	Workpiece material	Stainless steel HRC52
Feed rate (mm/min)	1555	Vibration sensor	Kistler 8636C acceleration sensor (Kistler, Winterthur, Swiss)
Tool feeding amount (mm)	0.001	AE sensor	Kistler 8152 acoustic emission sensor (Kistler, Winterthur, Swiss)
Cutting width (mm)	0.125	Force sensor	Kistler 9265B dynamometer (Kistler, Winterthur, Swiss)
Cutting depth (mm)	0.2	Data acquisition card	DAQ NI PCI 1200 (National Instruments, Austin, TX, USA)
Cutting length (mm)	108	Charge amplifier	Kistler charge amplifier (Kistler, Winterthur, Swiss)
Sampling frequency (kHz)	50	Microscope	LEICA MZ12 microscope (Leica, Solms, Germany)

**Table 2:** Hyperparameter settings.

Parameter	Learning Rate	Batch Size	Dropout Rate	Epoch	Optimizer	Activation
Value	0.001	128	0.2	500 (early stopping)	Adam	ReLU

**Table 3:** Model predictive performance with different input paradigms on three test sets.

Input Paradigms	Test Data						Computational Time (s)
	C1		C4		C6		
	MAE	RMSE	MAE	RMSE	MAE	RMSE	
downsampling-100	7.49	9.99	10.66	14.56	9.26	11.96	93.26
periodic-1	9.03	11.87	11.30	16.53	7.76	10.33	108.12
subsequence-300	7.10	8.92	8.92	11.39	10.88	13.78	76.89

**Table 4:** Model predictive performance of three input paradigms with different input sequence lengths on three test sets.

Input Paradigms	Input Sequence Length	Test Set					
		C1		C4		C6	
		MAE	RMSE	MAE	RMSE	MAE	RMSE
Downsampling paradigm	30	9.18	11.87	11.33	16.44	7.00	9.25
	150	6.73	8.95	10.59	15.92	10.57	13.46
	300	7.49	9.99	10.66	14.56	9.26	11.96
	1500	8.40	10.59	9.81	14.85	9.02	11.81
Periodic paradigm	30	9.94	13.89	13.54	20.17	12.24	16.76
	150	9.50	12.45	11.33	16.67	8.72	12.05
	300	9.03	11.87	11.30	16.53	7.76	10.33
	1500	9.99	13.40	11.42	16.54	11.08	14.27
Subsequence paradigm	30	5.87	7.43	10.35	12.50	11.16	14.42
	150	5.64	7.06	9.89	12.57	9.20	11.48
	300	7.10	8.92	8.92	11.39	10.88	13.78
	1500	6.86	8.64	8.29	12.20	9.66	12.79

**Table 5:** Model predictive performance with different subsequence numbers on three test sets.

Subsequence Number	Test Set					
	C1		C4		C6	
	MAE	RMSE	MAE	RMSE	MAE	RMSE
30	5.87	7.43	10.35	12.50	11.15	14.42
60	5.61	6.89	7.60	9.83	10.59	14.17
90	5.52	7.40	8.10	11.94	10.35	13.29
120	5.53	6.93	7.70	10.10	8.66	11.84
150	5.64	7.06	9.89	12.57	9.20	11.48
180	5.83	7.25	9.54	12.58	9.77	13.06
210	6.35	8.01	7.54	10.28	12.22	16.39
240	5.90	7.40	8.71	11.44	10.31	13.83
270	7.00	8.67	8.77	12.32	11.64	15.63
300	7.10	8.92	8.92	11.39	10.88	13.78

**Table 6:** Model predictive performance with different temporal indicators on three test sets.

Temporal Indicator	Test Set						Computational Time (s)
	C1		C4		C6		
	MAE	RMSE	MAE	RMSE	MAE	RMSE	
Maximum Values	5.53	6.93	7.70	10.10	8.66	11.84	43.00
Minimum Values	11.52	14.94	8.86	11.54	12.07	16.81	44.99
Mean Values	18.56	25.22	14.30	22.18	7.44	10.51	54.17

**Table 7:** Comparison of the method performance.

Method	Feature Engineering	Test Data					
		C1		C4		C6	
		MAE	RMSE	MAE	RMSE	MAE	RMSE
LR [49]	Yes	24.4	31.1	16.3	19.3	24.4	30.9
SVR [68]	Yes	10.1	13.12	13.69	15.4	12.42	14.74
SSAE-SVR [68]	Yes	8.6	11.89	11.52	14.75	8.25	10.95
CNN [69]	Yes	9.31	12.19	11.29	14.59	34.69	40.48
LSTM [54]	No	22.3	22.7	18.7	19.4	27.1	29.2
BiLSTM [54]	No	12.8	14.6	10.9	14.2	14.7	17.7
CNN-LSTM [70]	No	11.18	13.77	9.39	11.85	11.34	14.33
CABLSTM [55]	No	7.47	8.17	\	\	\	\
Proposed method	No	5.53	6.93	7.70	10.10	8.66	11.84

## Data Availability

PHM 2010 is available at https://phmsociety.org/phm_competition/.2010-phm-society-conference-data-challenge (accessed on 11 July 2022).
